# Accuracy and safety of robotic navigation-assisted distraction osteogenesis for hemifacial microsomia

**DOI:** 10.3389/fped.2023.1158078

**Published:** 2023-05-09

**Authors:** Ziwei Zhang, Zhijie Zhao, Wenqing Han, Byeong Seop Kim, Yingjie Yan, Xiaojun Chen, Li Lin, Weimin Shen, Gang Chai

**Affiliations:** ^1^ Department of Plastic and Reconstructive Surgery, Shanghai Ninth People's Hospital, Shanghai JiaoTong University School of Medicine, Shanghai, China; ^2^ Department of Burn and Plastic Surgery, Children's Hospital of Nanjing Medical University, Nanjing, China

**Keywords:** robot-assisted surgery, intelligent navigation, hemifacial microsomia, distraction osteogenesis, accuracy

## Abstract

**Introduction:**

This study aimed to verify the accuracy and safety of distraction osteogenesis for hemifacial microsomia assisted by a robotic navigation system based on artificial intelligence.

**Methods:**

The small sample early-phase single-arm clinical study, available at http://www.chictr.org.cn/index.aspx, included children aged three years and older diagnosed with unilateral hemifacial microsomia (Pruzansky-Kaban type II). A preoperative design was performed, and an intelligent robotic navigation system assisted in the intraoperative osteotomy. The primary outcome was the accuracy of distraction osteogenesis, including the positional and angular errors of the osteotomy plane and the distractor, by comparing the preoperative design plan with the actual images one week postoperatively. Perioperative indicators, pain scales, satisfaction scales, and complications at one week were also analyzed.

**Results:**

Four cases (mean 6.5 years, 3 type IIa and 1 type IIb deformity) were included. According to the craniofacial images one week after surgery, the osteotomy plane positional error was 1.77 ± 0.12 mm, and the angular error was 8.94 ± 4.13°. The positional error of the distractor was 3.67 ± 0.23 mm, and the angular error was 8.13 ± 2.73°. Postoperative patient satisfaction was high, and no adverse events occurred.

**Discussion:**

The robotic navigation-assisted distraction osteogenesis in hemifacial microsomia is safe, and the operational precision meets clinical requirements. Its clinical application potential is to be further explored and validated.

## 1. Introduction

Hemifacial microsomia (HFM) is one of the most common congenital craniofacial anomalies after cleft lip and palate, with an incidence of approximately 1/5,600 to 1/3,000, mostly disseminated (1). Its etiology is still unclear, which might be a combination of genetic and environmental factors (1). The most significant deformity of hemifacial microsomia is hypoplasia of the affected mandible, combined with hypoplasia of adjacent tissues. It can involve several extracranial systems, such as circulatory, respiratory, genitourinary, and skeletal systems, to varying degrees (1, 2). Patients often require early multidisciplinary intervention based on the skeletal treatment of the mandible.

Since 1992, when McCarthy et al. reported the successful treatment of HFM patients with mandibular distraction osteogenesis (MDO), it has become an essential tool in treating HFM (3). MDO not only effectively lengthens the mandibular ramus but also simultaneously lengthens the affected soft tissues, periosteal muscles, intraosseous vessels, and nerves (4). However, it is not easy to perform accurately, and the postoperative outcome depends on the precise operation. In particular, the narrow field of view makes preoperative planning difficult to implement precisely when the intraoral incision is used for esthetic purposes of avoiding extensive facial scars. Once the osteotomy line is skewed, it may lead to severe complications such as multiple osteotomy planes, dental germ damage, or rupture of the inferior alveolar neurovascular bundle. Additionally, the deviated placement of the distractor could lead to insufficient bone lengthening in the vertical vector, making it difficult to correct the occlusal plane. Therefore, maxillofacial surgeries such as MDO are often difficult to perform exactly according to the preoperative plans and are prone to unsatisfactory postoperative outcomes (5).

The continuous development of computer-assisted surgery (CAS) has laid the foundation for further improvement of surgical precision, assisting in the preoperative design, intraoperative navigation, and postoperative evaluation, and has been applied in craniomaxillofacial surgery with great clinical value (6). Moreover, with the integration of technological developments in various fields, various navigation technologies are equipped with intelligent robotic platforms, making surgical navigation more compatible with other imaging materials. Specialized medical navigation is also becoming miniaturized, specialized, systematic, automated, and noncontact. At present, static navigation are limited in materials, mostly plastic, which is not conducive to intraoperative drilling or osteotomy. In addition, there are safety hazards such as *in vivo* residue. Surgical robots have provided new ideas to improve the inherent limitations of static navigation in craniofacial surgery.

The electromagnetic (EM) navigation system is one of the novel navigation systems in clinical practice, consisting of a magnetic field generator, sensor interface units, and a control unit. It is able to track objects in real time and has the advantages of small size and easy installation (7). The registration accuracy is not limited by the light or soft tissue shifting. Therefore, EM navigation has broad application prospects. There has yet to be a mature, intelligent robotic navigation assistance system widely used for MDO. Based on previous studies, this study explored the accuracy of an intelligent planning-based robotic navigation system to assist in HFM distraction osteogenesis (8–11). Meanwhile, we evaluated the system's safety and explored its clinical application potential.

## 2. Materials and methods

### 2.1. Study design and clinical subjects

This study was an exploratory clinical trial of the application of cutting-edge technology and included a small sample of unilateral hemifacial microsomia patients from February 2022 to December 2022 for a single-arm trial. The inclusion criteria were hemifacial microsomia children with a three-dimensional computed tomography (3D-CT) diagnosis of Pruzansky type II mandible (type IIa: mandible with ramus, condyles, and temporomandibular joints present but hypoplastic and abnormally shaped; IIb: mandible with ramus hypoplastic and significantly abnormal shape and position, centered or anterior. The mandible is not articulated with the temporal bone). Exclusion criteria were (1) children younger than 3 years old; (2) diagnosis of Pruzansky type I (all structures of the mandible and temporomandibular joint present, normally shaped, varying degrees of dysplasia) or type III [ramus, condyle, and temporomandibular joint absent. The extensor pterygoid and temporalis muscles (if present) are not attached to the residual mandible] or other syndromes; (3) the patient has not given informed consent; (4) other congenital disorders are combined; (5) any contraindications to general anesthetic surgery.

This study was approved by the Independent Ethics Committee of Shanghai Ninth People's Hospital (SH9H-2021-T461-2) and followed the CONSORT guidelines. Patients and their guardians were fully informed, and written consents were obtained.

### 2.2. Preoperative design and system preparation

The robotic navigation system consisted of a computer base, a robotic arm (UR5), end-effectors, surgical navigation software, and an electromagnetic navigation system (Aurora V3, NDI). The system provided real-time navigation for the surgeon intraoperatively based on the preoperative plan with the corresponding end-effectors and presented the surgical plan in a visual interface that the surgeons could confirm at any time. The system had achieved real-time registration, recognizing the position movement of the surgical object. Once the system confirmed the change, the robotic arm could be adjusted to make the change to ensure that the design was accurately transferred into the actual operation.

**Figure 1:**
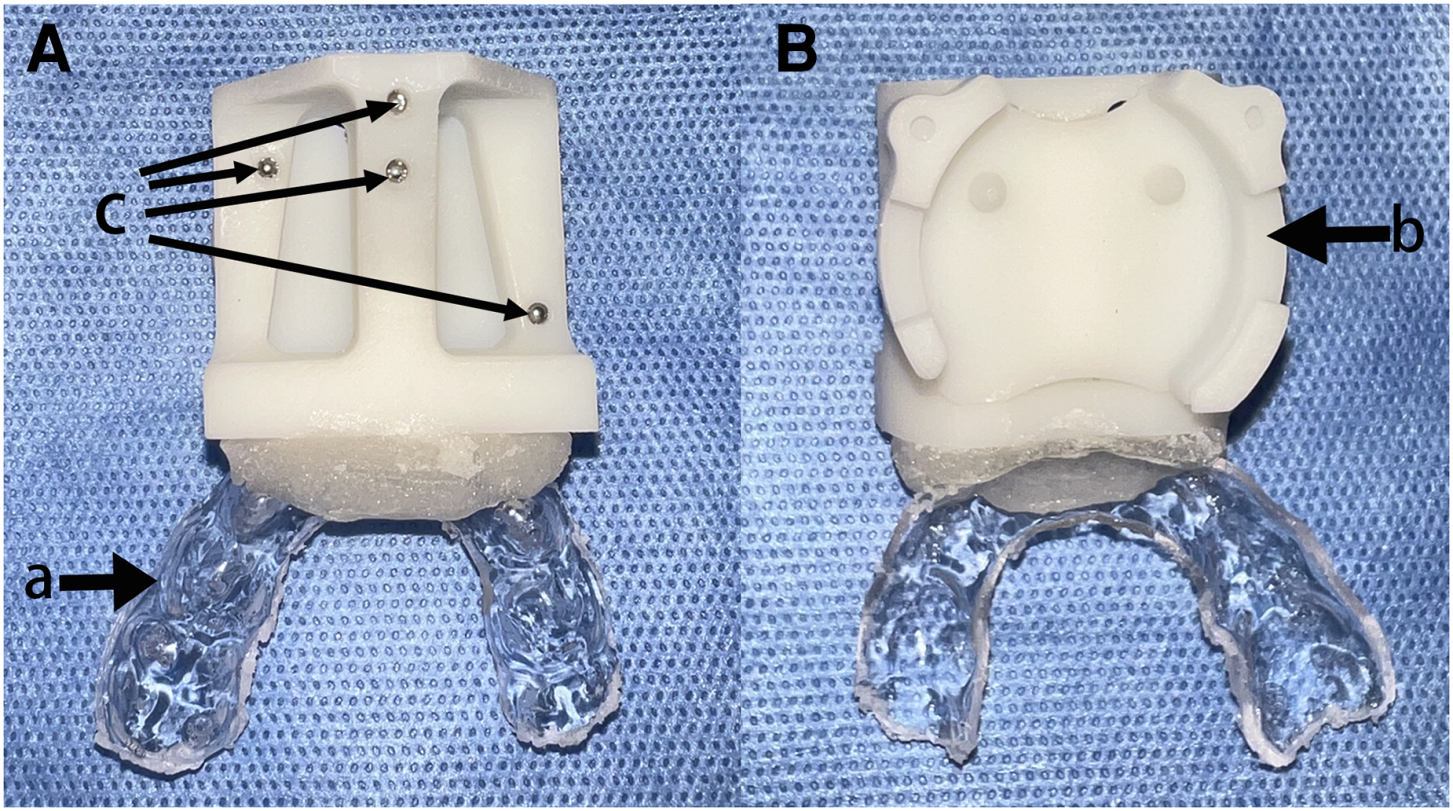
Registration piece. (**A**) Dorsal view. (**B**) Ventral view. (a) occlusal fixation part, (b) receptor base, and (c) attached steel beads.

For the system registration, the study used an occlusal registration piece for children (Figure 1). Four steel beads with a diameter of 2 mm were glued to the registration piece as positioning beads. On the other side, a receptor base was connected to the electromagnetic receptor.

**Figure 2:**
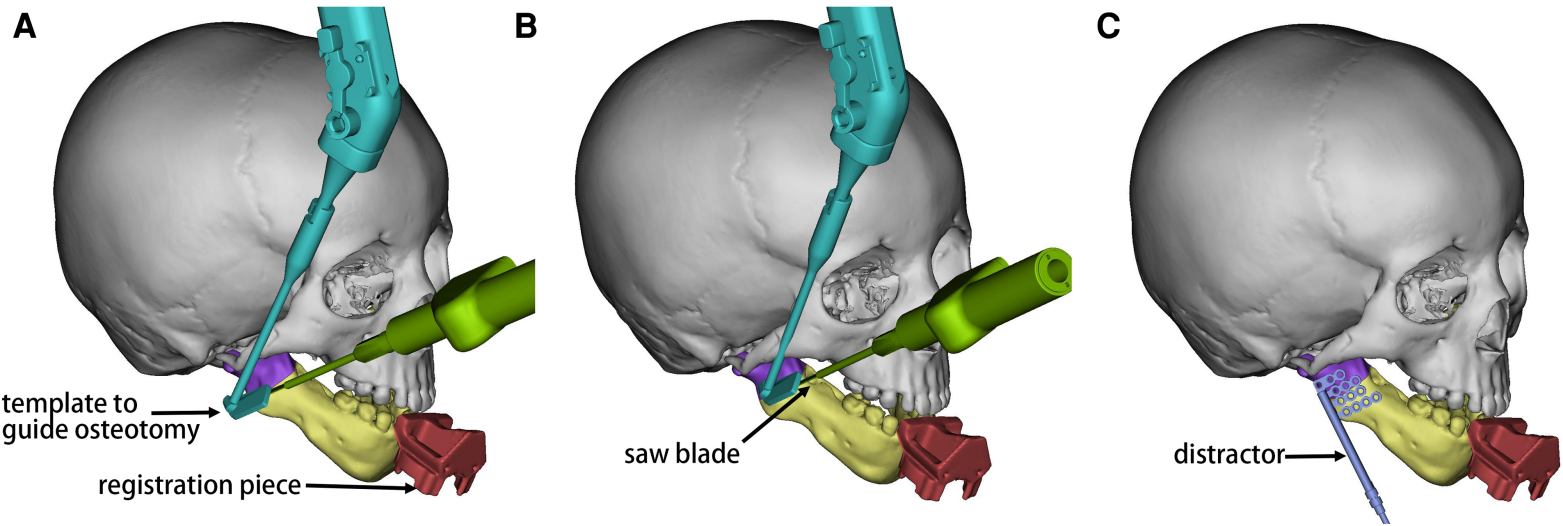
Schematic diagram of the preoperative design in Mimics. The mandibular ramus proximal to the osteotomy line is shown in purple and the distal part in yellow. (**A**) The end-effector (cyan) was in the initial position. The saw blade (green) was positioned along for the osteotomy operation. (**B**) The guide was moved to the second position, and the saw blade moved forward. (**C**) The position of the distractor (blue) was simulated with reference to the osteotomy line. The distraction direction was perpendicular to the osteotomy line, and sufficient bone was reserved for stabilizing the distractor.

Clinical standardized photographs were taken at the initial consultation, and a thorough extracranial system examination was performed. Particular attention was paid to checking oral hygiene for early prevention and treatment of dental caries. Enrolled patients received a full cranial 3D-CT scan, wearing the customized registration piece. The image data were imported into Mimics 21.0 (Materialise, Leuven, Belgium) in Digital Imaging and Communication in Medicine (DICOM) format. After the reconstruction, a preoperative design plan was completed by considering the mandibular deformity, occlusal plane, dental germ, and inferior alveolar nerve position. The plan includes the position of the osteotomy plane and the distractor, the direction, and the expected length of distraction (12). The spatial position of the surgical instruments, the end-effector, and the registration piece in the operative field should also be taken into account (Figure 2). The image data and design plan were saved in STL format and imported into the navigation system.

### 2.3. Registration and assisted distraction osteogenesis

**Figure 3:**
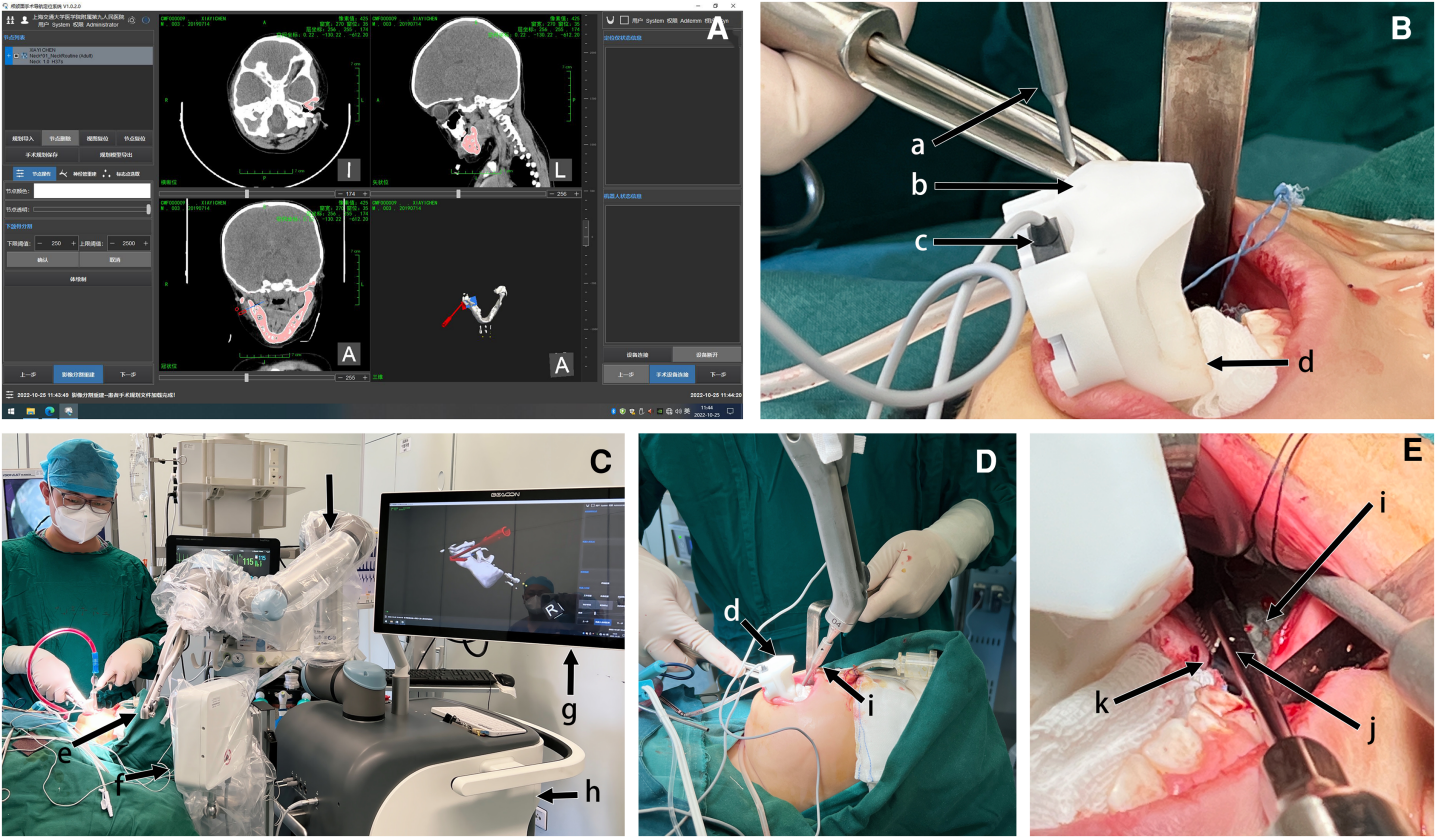
Robotic-assisted osteotomy procedure after registration. (**A**) Software screenshot showing the automatic recognition of the bead for registration and the planning of the guide path according to the preoperative design. (**B**) Position verification after registration by the navigation system. Pin tip movement to the set position of the registration piece indicated accurate registration. (**C**) Panoramic view. Open the “free movement” of the robotic arm to the vicinity and replace the end-effector of the osteotomy guide. (**D**) The robotic arm moved into place, and the guide moved to the osteotomy line position (extraoral view). (**E**) The saw blade was against the guide, and the osteotomy was performed from posterior to anterior. According to the generated path, the robot moved continuously from the first to the second position along the osteotomy line. The saw blade moved forward with it (intraoral view). (a) verification pin; (b) verification point; (c) electromagnetic receptor; (d) registration piece; (e) robotic arm end (end-effector not attached); (f) electromagnetic generator; (g) navigation system interface; (h) robot base; (i) osteotomy guide; (j) saw blade; (k) affected mandibular ramus.

After stripping the soft tissue on the mandibular ramus and angular area on the affected side was completed, the sterilized occlusal registration piece was appropriately worn on the patient's lower dentition. The system registration was then performed, i.e., the alignment of the image data with the physical mandible was accomplished by identifying the steel balls. The verification pin was attached to the robotic arm, and the operating interface executed the verification command. The pin tip was pointed directly above the set point on the registration piece, indicating an accurate registration (Figures 3A,B).

The system automatically performed the path design based on the imported preoperative design confirmed by the surgeons (Figure 3C). The end-effector was replaced with the osteotomy guide. “Free mode” was turned on, and the surgeon dragged the robotic arm to a position nearby. The end moved to the planned osteotomy on the Execute Path command (Figure 3D). The procedure requires an adequate stretch of the surrounding soft tissue to avoid injury. The surgeon held the saw with the blade relying on the guide, and moved slowly with it to complete the osteotomy (Figure 3E). Attention should be paid to the depth of the osteotomy to avoid damage to the inferior alveolar neurovascular bundle.

The built-in distractor was placed through the intraoral incision. The adjustment bar was passed through the submaxillary auxiliary incision, and the transbuccal instrument was placed through the buccal auxiliary incision. The endoscope was used to confirm the position of the distractor. Then, the distractor fixation was initially completed through the transbuccal instrument. Finally, a medial osteotomy of the ramus was performed until the mandible was utterly disconnected. Careful attention should be paid to the entire operation, and sutures should be placed after confirming adequate hemostasis. A negative pressure drainage tube was left in place with a pressure dressing.

### 2.4. Postoperative care and follow-up

Postoperatively, patients and guardians were given adequate rehabilitation education, and patients were instructed to pay attention to oral cleanliness. Patients underwent another cranial 3D-CT 1 week after the surgery to assess the accuracy of the procedure. Patient pain scales, satisfaction scales, and any occurred adverse events were also collected. Perioperative indicators were accurately recorded according to surgical documents.

### 2.5. Outcomes

**Figure 4:**
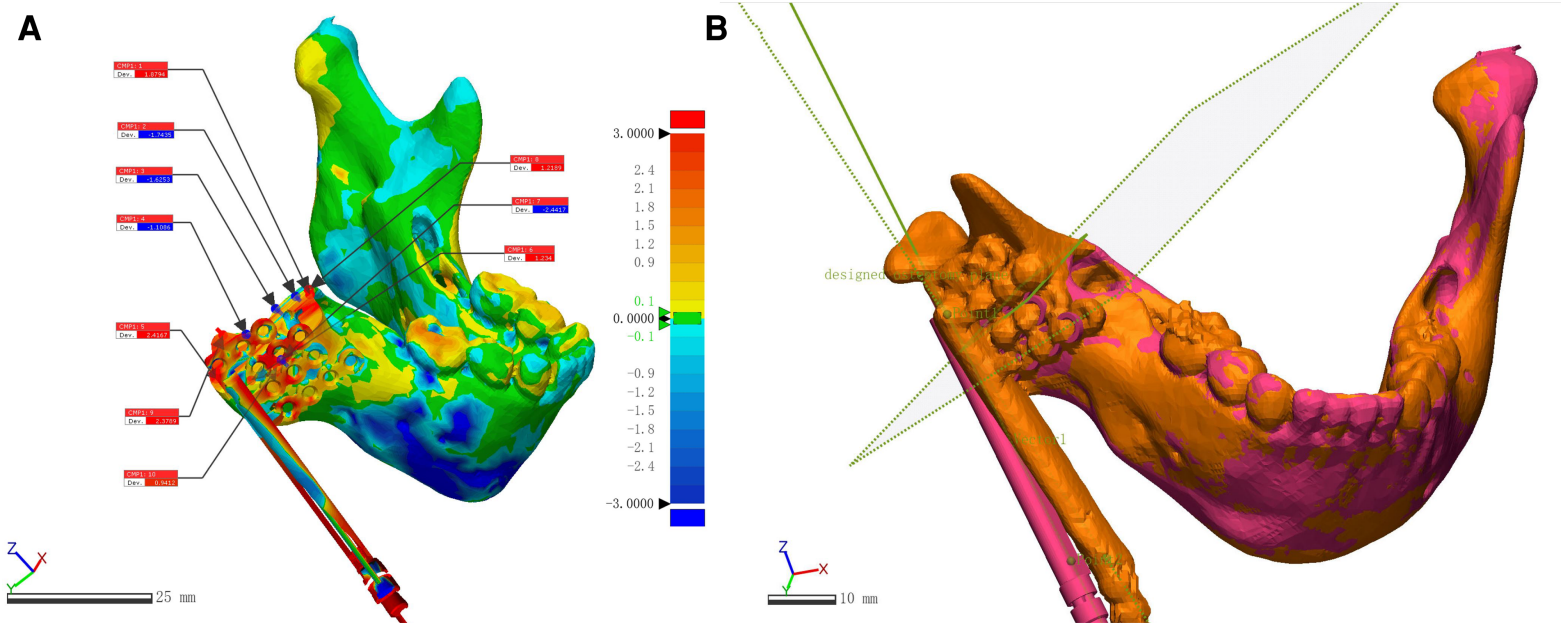
Accuracy analysis comparing preoperative design and postoperative CT. (**A**) Ten points were randomly selected on the osteotomy plane after automatic fitting to evaluate the positional error of the osteotomy. (**B**) The angular error of the osteotomy plane was measured by extracting the preoperative design and the postoperative osteotomy surface, respectively; the distance between the upper and lower end points of the distractor fixation bar was measured to evaluate the positional error; the angle between the vectors of the distractor fixation bar was measured to evaluate the angular error.

The primary outcome of this study was the operation's accuracy, which was measured by fitting the preoperative design to the actual CT data 1 week postoperatively. In the 3D reconstruction software (Geomagic ControlX, 2020, 3D Systems, Rock Hill, SC, United States), the two mental foramina and the highest point of the healthy condyle were selected as the alignment points. In the fitted 3D images, 10 points on the osteotomy plane were randomly selected as the positional error analysis points. The average of the absolute value of the distance error of these 10 points was used to evaluate the overall distance error of the osteotomy plane (Figure 4A); the preoperative design and the postoperative osteotomy plane were extracted, and the angle of these two planes was used as the angular error. The upper and lower endpoints of the distractor fixation bar were selected, and the line between the two points was the vector of the distraction. The positional error of the distractor was the average distance error of the upper endpoint and the lower endpoint of the fixation bar; the angular error of the distractor was the angle between the vectors of the two directions (Figure 4B). Each group of measurement data was taken by the same person who was not involved in the operation, and the data of each patient was measured three times. The average value was taken as the measurement data.

The secondary outcomes were (1) perioperative indicators, including operative time, hospitalization, operative bleeding, drainage time, and drainage volume; (2) pain scale (the pain scale is an internationally used visual analog scale from 0 to 10 representing no pain to the most intense pain, respectively); (3) complications; (4) satisfaction scale (postoperative satisfaction is a modified 5-point Likert scale from 1 to 5 representing dissatisfied to very satisfied, respectively).

### 2.6. Statistical analysis

Data measurements were performed by third-party personnel to assess the procedure's effectiveness. Data were analyzed by SPSS 26.0 (IBM Corp., Armonk, NY, United States). Quantitative data were counted and analyzed as mean ± standard deviation (SD).

Accuracy results were compared with results from previous preliminary animal experiments *via t*-test (osteotomy positional error: 2 mm; osteotomy angular error: 10°; distractor positional error: 5 mm; distractor angular error: 7°) (13). *α* was set as 0.05 and (1 − *β*) as 0.9. There was a statistical difference when *p* < 0.05.

## 3. Results

A total of four patients with unilateral HFM (age range, 3–14 years; average, 6.5 years) were included between February 2022 and December 2022. The male-to-female ratio was 1:1. Baseline demographic characteristics are described in Table 1. All patients completed a 1-week postoperative follow-up.

**Table 1:** Baseline characteristics.

No	Gender	Age	Affected side	Pruzansky–Kaban classification
1	Female	4	Right	M2b
2	Female	14	Right	M2a
3	Male	5	Right	M2a
4	Male	3	Right	M2a

The preoperative design was fitted to the postoperative mandible and a three-dimensional color deviation map was generated (Figure 4A). Measurements of positional and angular errors of the osteotomy planes are shown in Table 2. Compared with the preoperative design reference, the osteotomy positional error was 1.77 ± 0.12 mm, and the angular error was 8.94 ± 4.13°. For the error of the distractor placed with reference to the osteotomy line, the position error was 3.67 ± 0.23 mm, and the angular error was 8.13 ± 2.73° (Table 2). Compared with our previous study, positional errors were lower (*t* = 3.894, *p* = 0.030 for osteotomy and *t* = 11.529, *p* = 0.001 for distractor fixation), while there were no significant differences in angular errors (*t* = 0.516, *p* = 0.641 for osteotomy and *t* = 0.977, *p* = 0.401 for distractor fixation).

**Table 2:** Operative positional and angular errors.

No	Osteotomy positional error (mm)	Osteotomy angular error (°)	Distractor positional error (mm)	Distractor angular error (°)
1	1.84	8.99	3.55	13.64
2	1.63	11.76	3.99	4.25
3	1.89	11.91	3.46	8.56
4	1.70	3.08	3.66	9.05
Mean ± SD	1.77 ± 0.12	8.94 ± 4.13	3.67 ± 0.23	8.13 ± 2.73

SD, Standard deviation.

The system assembly time was approximately 20 min per patient before surgery. The mean operative time for the four patients was 185 ± 33.9 min, with intraoperative bleeding of 62.5 ± 25 ml. Negative pressure drainage was retained for 3 days postoperatively in all patients, with significantly more drainage in older patients. Patients were hospitalized for slightly longer than 1 week and were discharged after a good recovery (Table 3).

**Table 3:** Perioperative indicators.

No	Age	Surgical time (min)	Blood loss (ml)	Drainage time (day)	Drainage volume (ml)	Hospitalization (day)
1	4	150	50	3	10.5	9
2	14	225	100	3	63	7
3	5	165	50	3	12	9
4	3	200	50	3	23	9
		185 ± 33.9	62.5 ± 25	3 ± 0	27.1 ± 24.5	8.5 ± 1

At the 1-week follow-up, patients or their guardians assessed the current postoperative stage with a high level of satisfaction (Table 4). Intraoral wound pain was minimal under resting conditions, with a slightly higher score for the most intense pain level within a week of recall (Table 4).

**Table 4:** One-week follow-up.

No	Satisfaction	Pain score (resting)	Pain score (extremum)
1	5	2	4
2	5	1	4
3	5	2	3
4	4	3	5
	4.8 ± 0.5	2 ± 0.8	4 ± 0.8

No postoperative complications such as local infection, bone discontinuity, scar growth, occlusal disorders, nerve damage, or dental germ damage were observed in patients (by patient complaints, postoperative physical examination, or imaging).

## 4. Discussion

In recent years, with the continuous iteration of surgical robotics, surgical robots have been used in multiple clinical fields and have the application capability to achieve accurate operations under complex paths in maxillofacial surgery. The existing maxilla-craniofacial surgical robots are mainly composed of a surgical planning system, surgical positioning and navigation system, robot control system, mechanical structure, and feedback system (9, 14). The magnetic navigation used in this study is an emerging navigation method that can detect the accurate coordinate position of the patient and surgical instruments in real time based on an electromagnetic field generator and receptors. It has the advantage of not being affected by the obstruction (15). The center has completed the independent research and development of the robotic positioning system with electromagnetic navigation and has successfully applied it in the mandibular model experiment, cranial model, and animal experiments to improve the accuracy of osteotomy, which has initially verified the feasibility of electromagnetic navigation technology combined with robot-assisted surgery, but further clinical validation is still needed (8–11, 16–19).

In this study, the mandibular osteotomy positional error was within 2 mm, and the angular error was about 10° compared to the ideal preoperative design. There are no clinical trials of similar navigation systems applied to MDO in pediatric patients. Through the review of the literature, the osteotomy errors and distractor placement errors of this study were similar to previous clinical studies applying static guides and met the clinical accuracy requirements (20). Compared to previous preclinical model experiments of intelligent navigation system-assisted HFM distraction osteogenesis (21), the error in this study increased, which may be due to a further restricted field of view in the clinical setting and the soft tissues around the operative area. More metal surgical instruments, anesthesia machines, etc., all impacted navigation accuracy. Compared to previous clinical trials with similar navigation systems for mandibular angle osteotomy, the accuracy was very close (19). It is noteworthy that the errors of the distractor placement were relatively large. This may be because the distractor was placed and fixed concerning the osteotomy line, a procedure that was susceptible to external forces under high-stress surgical conditions and because the surgeon's hand–eye error could further amplify the errors of the previous procedure. Improvements in the existing navigation system are expected to further reduce overall surgical error by aiding distractor fixation, reducing the possibility of damage to the dental germ from the fixation screw, and achieving a more desirable lengthening result later.

No adverse events were observed, and based on clinical experience, there was no significant increase in operative time or bleeding and no prolongation of the patient's hospitalization. These results initially validated the feasibility and safety of the robotic navigation system-assisted distraction osteogenesis in HFM. Postoperatively, the patient did not experience severe pain and performed satisfactorily, although a longer follow-up is needed for further observation and evaluation.

The occlusal piece used for registration in this study is noninvasive, small in size, and customized with an occlusal fixation part that keeps the relative position of the steel balls and the mandible constant during the operation. With the real-time registration technique of the robotic navigation system, the moving mandibular position was recorded and fed back to the robotic arm for adjustment. However, this registration technique based on oral occlusal fixation only applies to patients with relatively well-grown teeth (including deciduous teeth) to perform the operation stably. If it is to be further promoted to expand the scope of the application, the application is limited to patients who need a distraction in infancy. With the development of CAS techniques, such as artificial intelligence algorithms and navigation recognition and positioning techniques, the team expects to develop registration and positioning based on appropriate soft tissue identification points.

The study has limitations. With a small sample size and a single-arm trial without controls, this study investigated the feasibility of intelligent navigation system-assisted MDO under clinical conditions to verify accuracy and safety initially. Demonstration of the improvement in accuracy remains subject to intergroup comparison with conventional surgery. The improvement of mandibular symmetry after complete distraction could also be evaluated during a longer-term follow-up to assess the postoperative outcomes. Also, the child's age and psychosocial development can be considered to improve the evaluation system.

The artificial intelligence-based robotic navigation system applied to HFM distraction osteogenesis could accurately translate the preoperative design intraoperatively and provide stable and reliable navigation support. The navigation system did not increase the original risk of the procedure and was safe. Further controlled studies with a large sample and long follow-ups were still needed to corroborate.

## Data Availability

The original contributions presented in the study are included in the article, further inquiries can be directed to the corresponding authors.
